# Impacts of hatchery-reared mandarin fish *Siniperca chuatsi* stocking on wild fish community and water quality in a shallow Yangtze lake

**DOI:** 10.1038/s41598-018-29758-z

**Published:** 2018-07-31

**Authors:** Wei Li, Brendan J. Hicks, Mingli Lin, Chuanbo Guo, Tanglin Zhang, Jiashou Liu, Zhongjie Li, David A. Beauchamp

**Affiliations:** 1 State Key Laboratory of Freshwater Ecology and Biotechnology, Institute of Hydrobiology, Chinese Academy of Sciences, Wuhan, 430072 China; 2 Washington Cooperative Fish and Wildlife Research Unit, School of Aquatic and Fishery Sciences, University of Washington, Box 355020, Seattle, WA USA; 3 Centre for Biodiversity and Ecology Research, Department of Biological Sciences, Faculty of Science and Engineering, The University of Waikato, Hamilton, New Zealand; 4 Institute of Deep-sea Science and Engineering, Chinese Academy of Sciences, Sanya, China; 5 U.S. Geological Survey, Western Fisheries Research Center, Seattle, WA USA

## Abstract

Mandarin fish *Siniperca chuatsi*, a valuable piscivorous fish, have been stocked into many lakes in China since the 1990s. This study did the first attempt to evaluate the ecological effects of hatchery-reared mandarin fish stocking in the Yangtze River basin lakes. Our study demonstrated a significant change in fish community composition after mandarin fish stocking, but no fish extinction was observed. No significant difference was observed in the total density of 13 forage fish before and after mandarin fish stocking, but the total biomass showed a significant decline after mandarin fish stocking. Significant differences in length-frequency distributions were observed for *Carassius auratus*, *Pseudorasbora parva* and *Toxabramis swinhonis* captured before and after stocking mandarin fish. No significant change in habitat distribution was detected before and after mandarin fish stocking. A marked decline in total nitrogen and a slight decline in total phosphorus were observed while a slight increasing trend for Secchi depth was found after stocking. Our findings suggested that mandarin fish stocking can increase predation pressure on forage fish and subsequently optimize the food web structure. Also, mandarin fish stocking has the potential to improve water quality and may be a feasible strategy to alleviate eutrophication of shallow Yangtze lakes.

## Introduction

Stock enhancement is a fisheries management approach involving the release of cultured organisms to increase abundance and yield of natural fish or invertebrate stocks^1^. In general, successful stock enhancement can improve socioeconomic outcomes by (1) creating new economic opportunities for fisheries-related livelihoods, (2) increasing skills of local communities, and (3) providing high-quality food fish and protein^2,3^. However, stocking or introduction of predators, especially piscivorous fish, may have strong impacts on freshwater ecosystems through predation, competition, mixing of exotic genes, habitat modification, and the introduction of pathogens^4–7^. When predation is intense, predators create both direct and indirect effects that cascade down the food web affecting both food web structure and biodiversity^8–12^. Direct predation on fish can change the species composition and size structure of the fish community at lower trophic levels and reduce population densities^13,14^. Indirect effects include habitat changes associated with predator avoidance behavior and increases of emigration rates of forage fish^13,15^. Therefore, understanding the changes on prey fish abundance and cascading effects down to lower trophic levels is essential for evaluating the ecological effects of stocking piscivorous fish in freshwater ecosystems.

Piscivorous fish stocking can be used as a lake restoration tool to improve water quality by creating a trophic cascade^16,17^. Direct predation on zooplanktivorous fish can initiate a trophic cascade that reduces predation pressure on zooplankton, especially on large cladocerans, leading to increased grazing on planktonic algae^18–20^. Predators may also reduce benthivorous fish, leading to a decrease in suspended material in the water column, thereby increasing Secchi depth^21^. Based on this evidence, stocking piscivorous fish in eutrophic lakes is commonly referred to as biomanipulation and is a possible strategy to alleviate eutrophication^19^.

Mandarin fish, or Chinese perch, *Siniperca chuatsi* (Basilewsky), is one of the most important commercial freshwater fish inhabiting Asian waters from the south in the Zhujiang River system to the north in the Amur River system^22^. The fish is a piscivore with specialized feeding habits, feeding only on live fish or shrimp throughout its life^23–25^. In the past several decades, mandarin fish populations in the Yangtze lakes have declined due to overfishing and habitat loss^25^. Thus, hatchery-reared mandarin fish have been stocked since the 1990s to increase populations^25^, to pursue commercial returns^26^, and to ease conflicts between fishery development and water quality conservation, based on the principle of trophic cascades^16^. By far, the area of lakes and reservoirs stocked mandarin fish exceeds 133000 ha in the middle and lower basins of the Yangtze River. The economic benefits of mandarin fish stocking in several lakes is well documented^26^, and studies on stocked mandarin fish have focused on survival, growth, recapture rate and foraging success^27–29^. In contrast, relatively little is known about the impacts of stocking mandarin fish on the ecology of Yangtze Lakes.

Stocked mandarin fish always incorporate both fish and shrimp into their diets^25^ and thus can alter the composition, densities, size distributions, and niche characteristics of wild fish populations directly or indirectly through predation or competition^6,15,19,30^. There is some evidence that stocked mandarin fish do not compete with wild *Siniperca* fishes during the critical periods of the early stocking stages^25^, but we know of no research that has explicitly focused on stocked mandarin fish impacts on wild fish community at lower trophic levels in lakes. To address this, we used a before-after study design to evaluate impacts of mandarin fish stocking on the wild forage fish community. Specifically, we compared the composition, abundance, size structure and spatial patterns of habitat use of wild forage fish before and after mandarin fish stocking in a shallow Yangtze lake, Central China. We predicted that mandarin fish stocking would significantly change the community structure of wild fish. Also, water quality was compared before and after mandarin fish stocking to evaluate whether piscivorous fish stocking can improve water quality through trophic cascade effects in shallow lakes along the Yangtze River.

## Results

### Mandarin fish density and biomass

After six years continuous hatchery-reared mandarin fish stocking, their density and biomass showed an apparent increase trend. The density and biomass of mandarin fish from 2012 to 2014 were significantly higher than those of 2009 before stocking (all P < 0.001, Table 1).

**Table 1:** Estimated mandarin fish density and biomass (mean ± SD) in Biandantang Lake from 2009 to 2014.

Year	Before stocking	After stocking				
	2009	2010	2011	2012	2013	2014
Density (individuals∙ha^−1^)	47.6 ± 7.1^a^	55.1 ± 7.7^a,b^	69.8 ± 10.8^b^	88.9 ± 10.7^c^	102.2 ± 10.4^c,d^	111.6 ± 14.6^d^
Biomass (kg∙ha^−1^)	16.2 ± 2.0^a^	17.0 ± 3.3^a^	19.5 ± 2.8^a^	24.9 ± 2.4^b^	29.0 ± 2.2^c^	32.7 ± 3.8^c^

For each row, means with different superscript letters are significantly different from each other (P < 0.05).

### Fish composition and abundance

A total of 19 fish species were collected by multiple mesh-sized gill nets from 2007 to 2014 in Biandantang Lake, of which 13 species were found in stomach contents of mandarin fish^25^. In 2007–2008, prior to the mandarin fish stocking, *Toxabramis swinhonis*, Sharpbelly *Hemiculter leucisculus*, *Culter dabryi*, crucian carp *Carassius auratus* and topmouth gudgeon *Pseudorasbora parva* were the dominant species in forage fish community, with an average numerical percentages of 24%, 21%, 14%, 10% and 10%, respectively. While in 2010–2014, after the mandarin fish stocking, *T*. *swinhonis* dominated the net samples with an average numerical percentage of 77%, followed by *H*. *leucisculus* (6%) and *C*. *dabryi* (6%). The percentage of *C*. *auratus* and *P*. *parva* declined gradually, and only accounted for 1.1% and 0.25% of the total number in 2014, respectively (Table 2). Although *T*. *swinhonis*, *H*. *leucisculus*, *C*. *dabryi*, *C*. *auratus* and *P*. *parva* were the most commonly captured forage fish before and after mandarin fish stocking in Biandantang Lake, the community composition of forage fish differed significantly between periods (χ^2^ = 63.65, df = 12, P < 0.001). Comparing the food habits of 13 forage fishes^31,32^, we found all 13 fish species feed more or less on zooplankton. Among them, the larvae of *C*. *auratus* and *C*. *dabryi*, and *P*. *parva* mainly feed on zooplankton (Table 2).

**Table 2:** Percentage of abundance in forage fish community before (2007–2008) and after (2010–2014) mandarin fish stocking in Biandantang Lake.

Forage fish species	Feeding habits	Percentage of abundance (%)						
		Before stocking		After stocking				
		2007	2008	2010	2011	2012	2013	2014
*Carassius auratus*	Small sized group (standard length (SL) < 50 mm) mainly feed on algae and zooplankton, middle group (5 mm < SL < 150 mm) mainly feed on zooplankton and macrophyte, and large group (SL >150 mm) mainly feed on zoobenthos^31^	9.53	10.61	4.40	3.12	2.16	0.88	1.01
*Culter dabryi*	Small sized group (SL < 100 mm) mainly feed on cladoceran and copepods, middle group (10 mm < SL < 200 mm) mainly feed on shrimps, large group (SL > 200 mm) mainly feed on small fish^31^.	13.50	15.10	9.86	11.75	3.14	3.18	3.91
*Toxabramis swinhonis*	Mainly feed on insect larvae, copepods and plant debris^32^.	22.59	24.49	60.21	71.90	82.99	87.95	81.69
*Hemiculter leucisculus*	Mainly feed on algae, followed by plant debris, small crustaceans and oligochaeta^31^.	22.86	18.61	7.39	5.51	5.88	3.84	9.46
*Pseudorasbora parva*	Mainly feed on copepods and cladoceran^31^.	10.41	9.06	4.23	1.93	1.42	0.71	0.25
*Rhodeus* spp.	Mainly feed on plant debris, followed by algae and zooplankton^32^.	7.06	6.94	3.96	0.37	0.00	0.00	0.00
*Rhinogobius giurinus*	Mainly feed on aquatic insect, followed by zooplankton and small fish^31^.	3.97	4.41	3.17	0.09	0.29	0.00	0.00
*Squalidus nitens*	Mainly feed on insect larvae, followed by plant debris and copepods^32^.	2.65	4.24	1.58	0.64	0.00	0.00	0.00
*Cultrichthys erythropterus*	Young fish feed mainly on cladoceran, copepods and aquatic insects, adult fish mainly feed on small fish and also feed on a small amount of aquatic insects shrimp and cladoceran^31^.	3.27	2.61	0.88	0.55	0.78	0.44	0.63
*Abbottina rivulars*	Mainly feed on cladoceran, copepods and amphipoda, followed by aquatic insect and plant debris^31^.	1.77	2.04	1.41	0.55	0.34	0.35	0.38
*Acheilogathus macropterus*	Mainly feed on algae and plant debris, followed by cladoceran^31^.	1.06	1.22	1.14	1.10	0.93	0.79	0.32
*Coilia brachygnathus*	Small sized group (SL < 250 mm) mainly feed on copepods, insect larvae, cladoceran and small fish, large sized groups (SL > 250 mm) mainly feed on small fish and shrimps^31^.	0.53	0.33	1.41	2.48	1.72	1.85	2.21
*Hyporhamphus intermedius*	Mainly feed on copepods and cladoceran, followed by aquatic insects^31^.	0.79	0.33	0.35	0.00	0.34	0.00	0.13

### Forage fish density and biomass

**Figure 1:**
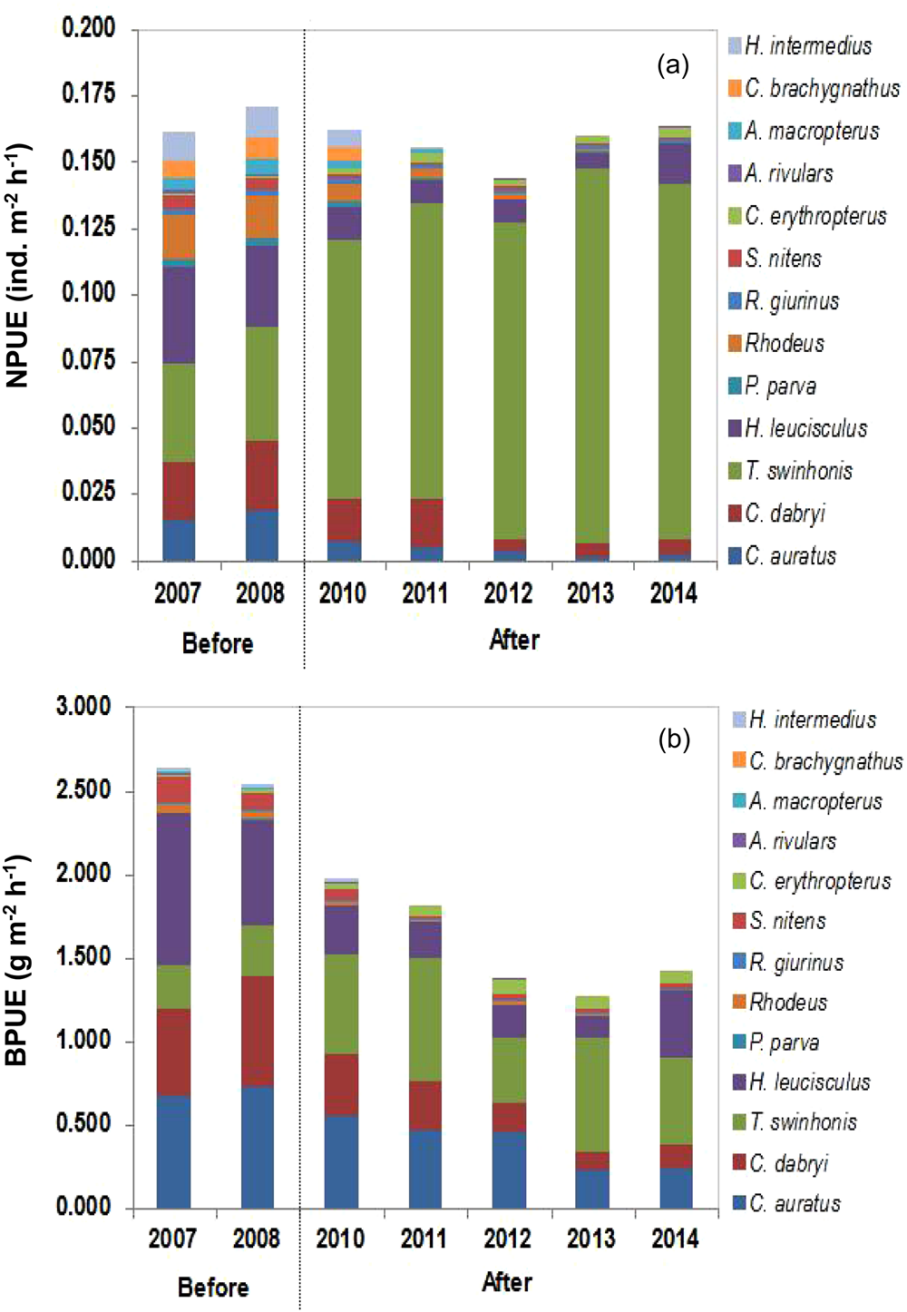
Changes in forage fish density (**a**) and biomass (**b**) before and after mandarin fish stocking in Biandantang Lake.

The total density of 13 forage fish ranged from 0.143 to 0.171 individuals∙m^−2^∙h^−1^ during our study period, and no significant difference was observed before and after mandarin fish stocking (Mann-Whitney test, P = 0.081) (Fig. 1a). However, there was a significant difference in the total biomass of 13 forage fish before (mean: 2.59 g∙m^−2^∙h^−1^) and after (mean ± SD: 1.57 ± 0.31 g∙m^−2^∙h^−1^) mandarin fish stocking (Mann-Whitney test, P < 0.001). The total biomass ranged from 1.275 to 2.637 g∙m^−2^∙h^−1^ and show an apparent declining trend after mandarin fish stocking (Fig. 1b). For several dominant forage fish, a significant decreased trend was observed in the density and biomass of *C*. *auratus*, *H*. *leucisculus*, *C*. *dabryi* and *Rhodeus* spp. following mandarin fish stocking (Mann-Whitney test, P < 0.001). In contrast, the density and biomass of *T*. *swinhonis* showed an apparent increase after mandarin fish stocking, respectively (Mann-Whitney test, P < 0.001) (Fig. 1).

### Size structure of forage fish

**Figure 2:**
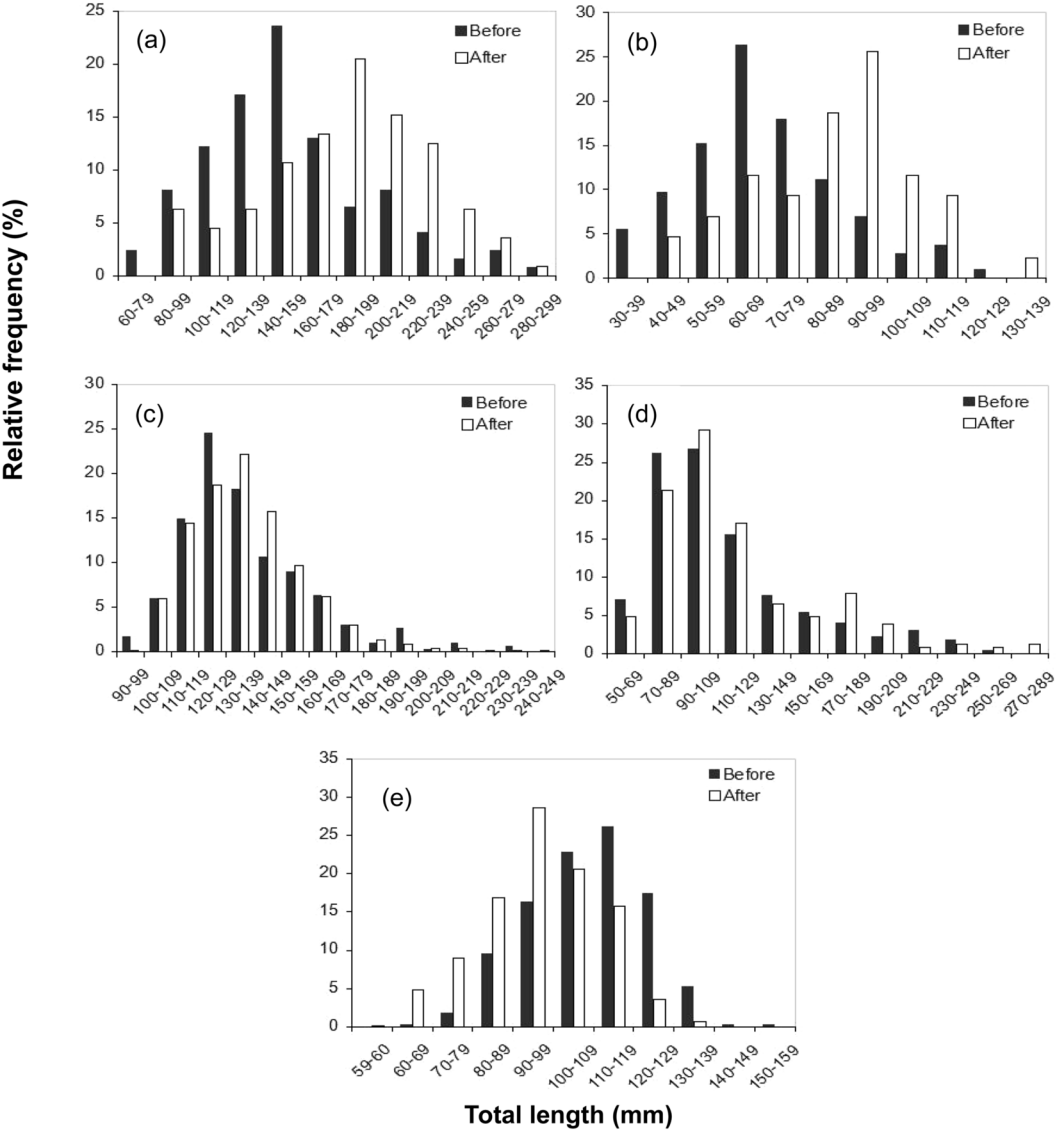
Total length frequency histograms for *C*. *auratus* (**a**) *P*. *parva* (**b**) *H*. *leucisculus* (**c**) *C*. *dabryi* (**d**) *T*. *swinhonis* (**e**) captured before (2007–2008, dark bars) and after (2010–2014, open bars) stocking mandarin fish in Biandantang Lake.

Significant differences in the length-frequency distributions were found for *C*. *auratus* (K-S test, D_max_ = 0.365, P < 0.001), *P*. *parva* (K-S test, D_max_ = 0.442, P < 0.001), and *T*. *swinhonis* (K-S test, D_max_ = 0.320, P < 0.001) captured before and after stocking mandarin fish in Biandantang Lake, but no significant difference was observed for *H*. *leucisculus* (K-S test, D_max_ = 0.034, P = 0.064) and *C*. *dabryi* (K-S test, D_max_ = 0.020, P = 0.103) (Fig. 2). Much larger *C*. *auratus* and *P*. *parva* and much smaller *T*. *swinhonis* were found in Biandantang Lake after mandarin fish stocking compared to before stocking. Indeed, mean total lengths of *C*. *auratus* and *P*. *parva* after mandarin fish stocking were significantly larger than those before stocking (t-test, *C*. *auratus*: t_234_ = −5.264, P < 0.001; *P*. *parva*: t_256_ = −5.403, P < 0.001), while mean total lengths of *T*. *swinhonis* were significantly small after mandarin fish stocking (t-test, t_2076_ = 18.426, P < 0.001) (Fig. 2).

### Habitat distribution

**Figure 3:**
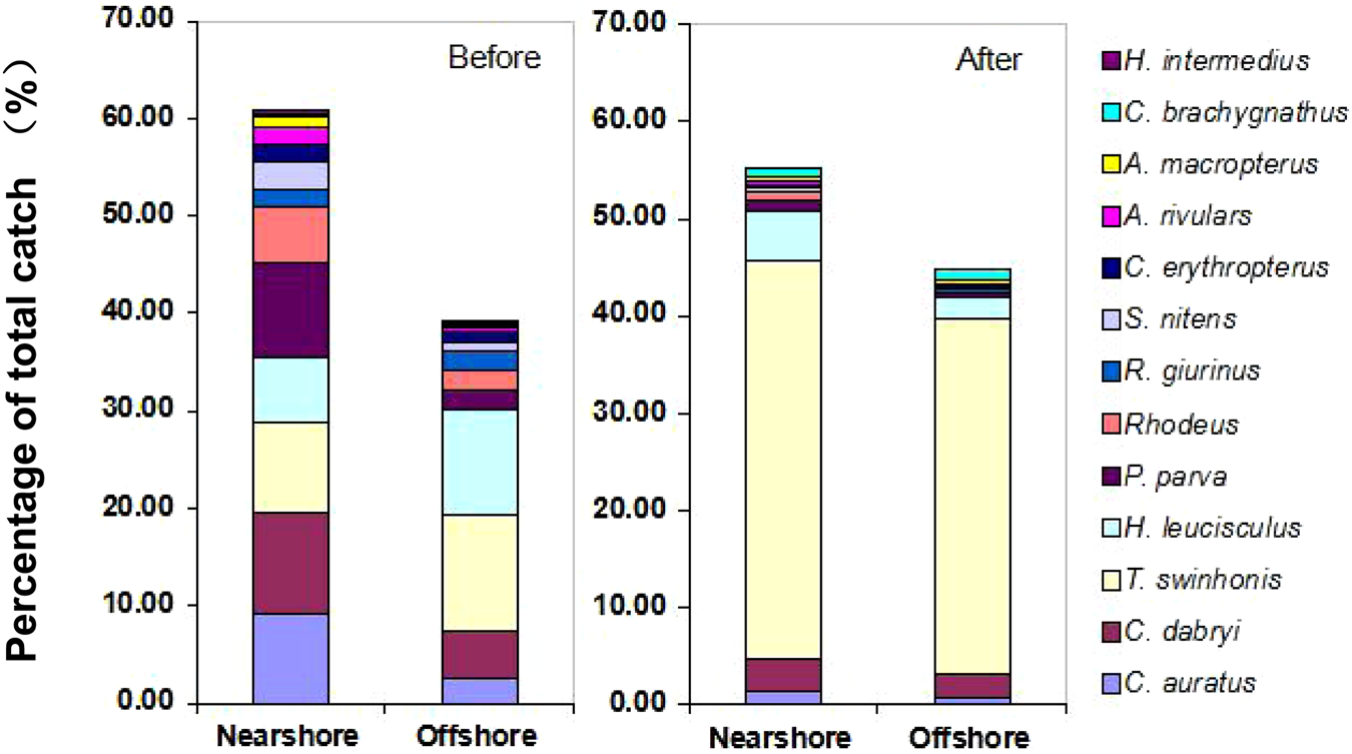
Mean (SD) percentages of total catch of forage fish at two different habitats (nearshore and offshore) before (2007–2008) and after (2010–2014) mandarin fish stocking in Biandantang Lake.

Both before and after mandarin fish stocking, forage fish were predominantly captured in nearshore habitat in similar proportions (percentage of total catch: before: $\bar{\chi }$ = 60.8 ± 1.3%; after: $\bar{\chi }$ = 54.3 ± 4.8%, Fig. 3). The percentages of forage fish were significantly higher nearshore than offshore during both periods (F = 35.424, P < 0.001), but those differences declined after stocking as indicated by significant interactions between treatment and habitat (F = 6.584, P = 0.028). For *C*. *auratus* and *C*. *dabryi*, the percentage in nearshore habitat was significantly higher than those in offshore habitat prior to stocking (*C*. *auratus*: F = 39.718, P < 0.001; *C*. *dabryi*: F = 32.559, P < 0.001), but no significant difference was observed after stocking (*C*. *auratus*: F = 0.078, P = 0.124; *C*. *dabryi*: F = 0.066, P = 0.298, Fig. 3). For *T*. *swinhonis* and *H*. *leucisculus*, the percentage in nearshore habitat was lower than in offshore habitat before stocking, but became higher nearshore than offshore after stocking. For *P*. *parva* and *Rhodeus* spp., the percentage nearshore was significantly higher than offshore both before and after mandarin fish stocking (before: *P*. *parva*, F = 45.114, P < 0.001; *Rhodeus*, F = 40.359, P < 0.001; after: *P*. *parva*, F = 23.581, P < 0.001; *Rhodeus*, F = 25.441, P < 0.001), but the ratio of the percentage in nearshore to offshore habitat before stocking was lower than after stocking (Fig. 3).

### Water quality

**Figure 4:**
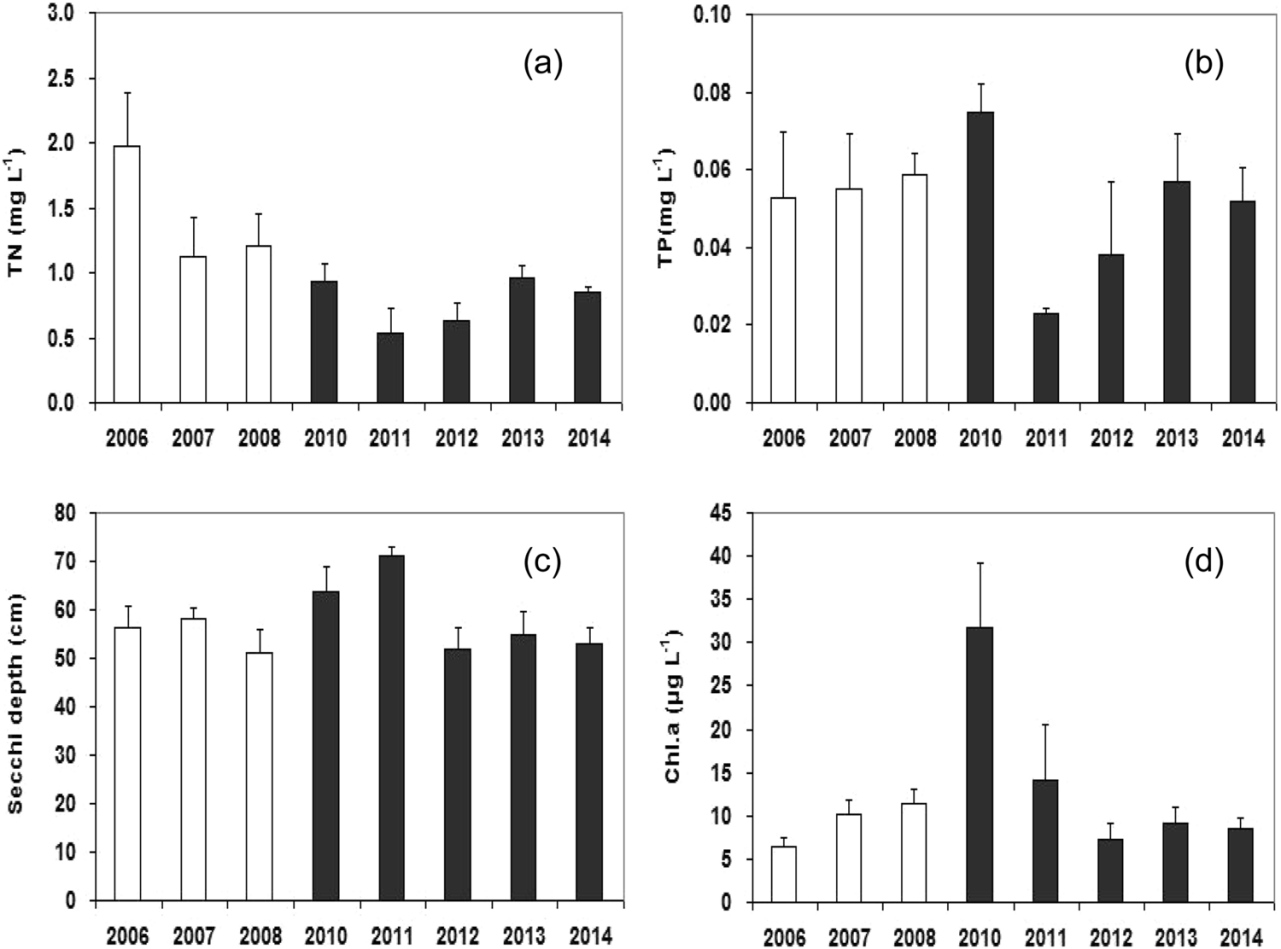
Changes in concentrations of (**a**) total nitrogen (TN), (**b**) total phosphorous (TP), (**c**) chlorophyll-*a* (Chl. a) and (**d**) Secchi depth before (open bars) and after (dark bars) mandarin fish stocking in Biandantang Lake.

The concentration of total nitrogen (TN) was significantly reduced after mandarin fish stocking (Mann-Whitney test, P < 0.001, Fig. 4a). Total phosphorus (TP) concentration showed more variability after stocking, but no apparent trend between mean values 0.056 mg L^−1^ during 2006–2008, to 0.048 mg L^−1^ during 2010–2014 (Mann-Whitney test, P = 0.349, Fig. 4b). Secchi depth was similar before and after mandarin fish stocking despite two years of improved visibility immediately following the start of stocking (2010 to 2011) (before: $\bar{\chi }$ = 55.2 ± 4.8 cm; after: $\bar{\chi }$ = 58.9 ± 8.6 cm. Mann-Whitney test, P = 0.370, Fig. 4c). Chl *a* concentration in 2010 increased immediately following stocking, but the means were not different before and after stocking (Mann-Whitney test, P = 0.410, Fig. 4d).

## Discussion

Apparent effects of top predators on lower trophic levels in freshwater ecosystems are common, and stocking or introductions of top predators into new ecosystems can reduce prey fish abundance and cause cascading effects through lower trophic levels^19,20,33,34^. Our study in Biandantang Lake demonstrated a significant change in forage fish community composition from one with similar proportions of *T*. *swinhonis*, *H*. *leucisculus*, *C*. *dabryi*, *C*. *auratus* and *P*. *parva* prior to mandarin fish stocking to a community dominated by *T*. *swinhonis* after stocking. The observation of reduced species diversity following stocking with a top piscivore is consistent with previous studies for piscivorous fish in North American and European countries^10,11,20,34^, but this is the first evidence that mandarin fish stocking exert strong effects on the community structure of wild forage fish in the shallow Yangtze lakes. The observed changes in forage fish community in our study was mainly embodied with the apparent difference in percentage of abundance of different forage fish, as no reduction of species richness of forage fish was observed after mandarin fish stocking. This was probably due to the wide food spectrum and selective predation of mandarin fish^24,25^.

Our before- and after-stocking comparative study provided no evidence that mandarin fish stocking reduced the total densities of wild forage fish populations, nor did the richness of wild forage fish decline, despite the well documented negative impacts that stocked piscivores have on many wild species^10,35^. Some factors could contribute to why piscivores stocking did not lead to reductions in total densities of forage fish: (i) feeding behaviour of predators, (ii) prey fish species and availability in environment, (iii) morphology and behavioural responses of forage fish, and (iv) habitat heterogeneity^15,35^. Unlike oligotrophic alpine lakes, mesotrophic or eutrophic shallow lakes of the Yangtze River basin including Biandantang Lake support an abundance of small-sized fish, which can serve as ample alternative prey for mandarin fish, who as a generalist predator can feed on a wide spectrum of available prey^24^. Over the initial five years after stocking mandarin fish, populations of these other forage fishes were reduced, but not extirpated. Therefore, the apparent increase of *T*. *swinhonis* population compensated for the reduction of other forage fish commonly consumed by mandarin fish, which accounted for the lack of a significant reduction in the total densities of forage fish after mandarin fish stocking.

In contrast to total densities, the total biomass of forage fish showed a significant reduction following stocking of mandarin fish. This reduction was apparently a result of both the direct effects of predation and indirect effects such as habitat shifts in an attempt to avoid predators, and a decrease in the average size of forage fish considered most vulnerable to predation^11^. The lack of trends in the total densities and biomass could be attributed to variations in community composition of forage fish. The reduction in abundance of relatively large-bodied forage fish species including *C*. *auratus*, *H*. *leucisculus* and *C*. *dabryi* directly caused the reduction of the total biomass of forage fish after mandarin fish stocking in Biandantang Lake.

For several dominant forage fish, their density in Biandantang Lake changed during the years of mandarin fish stocking in accordance with the increased predation pressure from stocked mandarin fish. The density of *C*. *auratus*, *H*. *leucisculus*, *C*. *dabryi* and *Rhodeus* declined by more than 50% after mandarin fish stocking. *Rhodeus* and *C*. *auratus* were affected more than the other two species, which can be explained by the difference of habitat choice. *Rhodeus* and *C*. *auratus* associate with the nearshore zones more than *H*. *leucisculus*, and *C*. *dabryi*^36^. Mandarin fish stocked in the nearshore zone occupied similar deeper depths and had easier access to *Rhodeus* spp. and *C*. *auratus* as a prey. In contrast, *H*. *leucisculus*, and *C*. *dabryi* occupied the upper layers of the water column further offshore, likely reducing their encounter rate with predators^31^. For *T*. *swinhonis*, one of the dominant forage fish in Biandantang Lake before stocking, its density increased dramatically following stocking of mandarin fish. *T*. *swinhonis* have a robust spiny dorsal fin, and thus greater anti-predation ability than the soft fin species^31,37^.

Apart from the stocked mandarin fish, other factors may cause the change in forage fish community composition after hatchery-reared mandarin fish stocking. Generally, temperature is often considered to be an important factor determining reproductive success and survival for fish^19,38^. Actually, no unusual weather conditions were occurred during the present study. In addition, the decline in biomass of forage fish might be caused by a fish-kill resulting from low oxygen concentrations, rather than by predation^19^. However, there is no direct evidence that such a fish kill observed. The fact that crucian carp, a species exceptionally tolerant to low oxygen concentrations, also declined, further indicates that the fish-kill hypothesis is unlikely.

Significant differences in the length-frequency distributions were observed for *C*. *auratus*, *P*. *parva*, and *T*. *swinhonis* captured before and after stocking mandarin fish. This effect was caused by size-selective predation of mandarin fish^24^. Size-selective predation commonly leads to increased mortality at early life stages of prey^24,39^ and can consequently alter the size structure of forage fish communities^37,40^. In this study, the mean total lengths of *C*. *auratus* and *P*. *parva* after mandarin fish stocking were significantly larger than those prior to stocking, suggesting that their populations are often dominated by larger size classes after mandarin fish stocking. The similar results were seen following stocking of other piscivorous fish, such as trout^15,41^. These results also indicated that more of the smaller *C*. *auratus* (120–159 mm) and *P*. *parva* (60–79 mm) were consumed with the increased density of mandarin fish. For forage fish *P*. *parva*, mandarin fish consistently preferred to consume individuals of 60–79 mm TL, despite consuming much larger topmouth gudgeon than expected from size distributions in Xiaosihai Lake near to Biandantang Lake^24^. This indicated that increased mandarin fish may cause increased mortality on 60–79 mm TL individuals of *P*. *parva* in the environment. For the forage fish *C*. *auratus*, disproportionate consumption of small individuals may result from gape limitations of mandarin fish, because crucian carp is a deep bodied fish and have the largest body depth of the five dominant forage fish over similar lengths. In addition, the mechanisms other than morphological limitations, such as greater abilities of larger individuals to escape predators, via spatial segregation or greater evasive capabilities, should also be taken into consideration. In contrast to *C*. *auratus* and *P*. *parva*, the mean total lengths of *T*. *swinhonis* were significantly smaller after stocking than before. The reduction in modal size and increased abundance was likely due to: (1) The spiny dorsal fin on *T*. *Swinhonis*, reduced the risk of occupying the same spatial niche as mandarin fish, as suggested by their low electivity as prey for mandarin fish^24,25^. (2) The food competition between *T*. *Swinhonis* and other zooplanktivores was relaxed substantially because of the reduced density of zooplanktivorous fish.

The nearshore-offshore habitat shifts by varied among species of forage fish after mandarin fish stocking, although no significant difference in the composite percentages of forage fish in nearshore versus offshore zones was observed. Generally, forage fish commonly occurred in or around nearshore zones with complex habitat as refuge and support more food resource^36,42,43^. We attribute the observed offshore shift by most species to increased predation risk in nearshore zones following stocking of mandarin fish which predominantly utilize nearshore habitats^31^. Typical nearshore forage fishes like *C*. *auratus*, *P*. *parva* and *Rhodeus* spp. shifted to offshore habitats after mandarin fish were stocked. However, *T*. *swinhonis* and *H*. *Leucisculus*, which were relatively more abundant in offshore habitat than in nearshore habitat before mandarin fish stocking, became the predominant forage fishes in both habitats after stocking. The inconsistent response to increased predation risk can possibly be ascribed to the different anti-predation ability among forage fish species. Although abundant food resources were available in nearshore zones, *C*. *auratus*, *P*. *parva* and *Rhodeus* spp. shifted offshore to escape predation. In contrast, with increased food competition offshore, *T*. *Swinhonis* and *H*. *Leucisculus* further exploited the nearshore zones to feed where the spiny dorsal fin of *T*. *Swinhonis* and faster swimming speeds of *H*. *Leucisculus* afforded greater anti-predation ability to mandarin fish than the other forage fishes^31^.

A marked decline in total nitrogen concentration and slight increase in Secchi depth were observed after mandarin fish stocking, suggesting that stocking mandarin fish has the potential to improve water quality of shallow Yangtze lakes. Our findings support Potthoff *et al*. opinion that stocking piscivorous fish can improve ecological characteristics of shallow lakes^20^. Generally, there was a positive relationships between chlorophyll-a concentration and concentrations of total nitrogen and total phosphorous^44–46^, however, the chlorophyll-a concentration in the study showed a slight but significant increase after mandarin fish stocking. This may have contributed to the increased density of *T*. *swinhonis* that feeds predominantly on insect larvae, copepods^31^. Although *P*. *parva*, juvenile *C*. *auratus* and *C*. *dabryi* also mainly feed on zooplankton^31^ and their reduced densities can reduce predation pressure on zooplankton, the significant increase in *T*. *swinhonis* may compensate for this reduced predation pressure and even intensify the pressure, and consequently cause the increase of phytoplankton through trophic cascade effects. Despite chlorophyll-a concentration showed a slight increase after mandarin stocking, Secchi depth also raised slightly. This is inconsistent with widespread agreement that Secchi depth generally negatively correlated with chlorophyll-a^47,48^. The inconsistence may be explained by the change of suspended solids. Previous study demonstrated that suspended solid is also an important factor influencing Secchi depth^21^. And the important effects of benthivorous fish on sediment resuspension has been frequently observed in other studies^21,49^. In this study, mandarin fish has specialized feeding habits of only feeding on live fish or shrimp throughout their life, and crucian carp, topmouth gudgeon, sharpbelly were the dominant food in their stomach in many lakes in the middle reach of the Yangtze River^24,25^. Although there is no direct evidence that suspended solids decreased in our study, the fact that reduced density of benthivorous *C*. *auratus* induced by increase predation pressure of mandarin fish after continuous stocking was able to diminish sediment resuspension in Biandantang Lake, seems possibly causing a resultant beneficial effect on Secchi depth.

Another experiment conducted in a highly eutrophic shallow Yangtze lake showed that the ecological status could be rapidly reversed from eutrophic to oligotrophic using an integrity measure combining a ban on fertilizer and a reduction of planktivorous fish stocking along with the introduction of both mandarin fish and Chinese mitten crab^50^. However, there is direct documentation that stocking piscivorous fish alone is useful for water quality protection in the shallow Yangtze lake, which means it could be a feasible bio-manipulation measure to control eutrophication for these lakes located in the middle and lower Yangtze River. Nowadays, water and food security have been and remain two of the most important priorities for the growing population of China. However, hundreds of lakes on the floodplain of the Yangtze River, like Lake Biandantang, are eutrophic and abundant with low value small-sized fish due to anthropogenic activities^26,51^. Although catchment-level restoration measures, especially nutrient abatement, were considered to have priority for shallow lake protection^52^, another study suggested that catchment-level restoration alone has limited potential to mitigate effects of eutrophication in shallow lakes^53^. So, managers as well as policy makers might consider using bio-manipulation based piscivorous fish stocking, in conjunction with catchment-level restoration measures, to stimulate improvement of ecological characteristics and development of sustainable fisheries in shallow Yangtze lakes.

In conclusions, this study is the first attempt to evaluating the ecological effects of stocking mandarin fish in shallow Yangtze lakes. This study supports the hypothesis that piscivorous fish may be a significant structuring force in shallow eutrophic lakes. Our results suggested that mandarin fish stocking have significant impacts on community composition, total biomass, size distribution and habitat use of forage fish by selective predation. Our data also indicated that stocking mandarin fish has the potential to improve water quality of shallow Yangtze lakes. As no significant difference in total density and no fish extinction was observed after mandarin fish stocking, it is suggested that mandarin fish stocking could be a feasible bio-manipulation measure, in conjunction with catchment-level restoration measures, to alleviate eutrophication for managers in shallow Yangtze lakes. However, the stocking balance needs to be carefully weighed as overstocking could have strong negative effects on fish community by replacing wild top consumers and changing ecosystem configuration through strong cascading effects on lower trophic levels. So, studies on optimizing stocking density and size of the stocked individuals and its long-term improvements should be taken into consideration in future.

## Materials and Methods

### Study site

**Figure 5:**
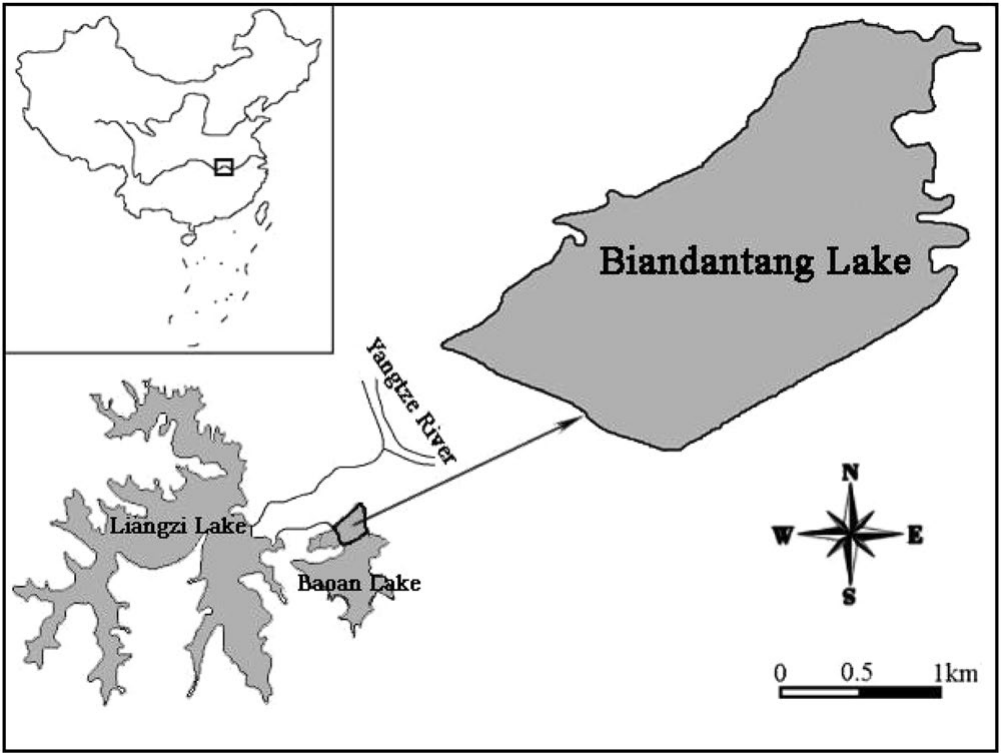
Map showing the geographic location and broad outline of Biandantang Lake. The figure was generated by the software Adobe Photoshop, version CS 6 (Adobe Inc., San Jose, California).

Biandantang Lake (30°15′N, 114°43′E) is located on the south bank of the middle reach of the Yangtze River, Hubei Province, Central China. The lake, with an area of 3.333 km^2^ and depths ranging from 1.2 to 3.2 m (mean 2.0 m), has been separated from the larger Baoan Lake by a dyke (Fig. 5). Fifteen years ago, Biandantang Lake was heavily covered with submerged macrophytes, with *Vallisneria spiralis* L., *Myriophyllum spicatum* L. and *Nelunbo nucefera* G. the dominant species^54^. For six years prior to this study, the major fishery had been supported by stocking Chinese mitten crab *Eroichier sinensis*. Subsequently submerged macrophytes, one of the most important food resources for *E*. *sinensis*, gradually decreased due to overstocking of *E*. *sinensis* in the past several years. During our study periods, the lake was only sparsely vegetated with *M*. *spicatum* and *Trapa bispinosa* Roxb in a small area of the littoral zone, and was slightly eutrophic. The fish community in the lake comprised 47 fish species belonging to 14 families, of which bighead carp *Aristichthys nobilis* (Richardson), common carp *Cyprinus carpio* (Linnaeus), silver carp *Hypophthalmichthys molitrix* (Valenciennes), crucian carp *Carassius auratus* (Linnaeus), *Culter dabryi* and mandarin fish were the most important commercial fish.

### Mandarin fish stocking

Each spring since 2009, Biandantang Lake has been stocked with juvenile hatchery-reared mandarin fish at densities of 14–120 individuals∙ha^−1^ (Table 3). The mandarin fish were hatched and raised in a nearby national fish hatchery. At an age of about 1–2 months, they were transported to the lake and released as much as possible over the littoral zone from a boat. In late June 2009, 4,800 juveniles (mean total length = 69.3 mm), marked with coded wire tags (tags with 0.25 mm diameter and 1 mm length, Northwest Marine Technology (NMT), Shaw Island, Washington) were stocked in order to evaluate the diet composition between hatchery-reared and wild mandarin fish^23^. From 2010 to 2014, juveniles were stocked in mid-June at a size of 25–35 mm total length.

**Table 3:** Number of mandarin fish stocked into Biandantang Lake from 2009 to 2014.

Date	Number (individuals)	Total length (mm)		Stocking density (individuals∙ha^−1^)
		Range	Mean ± SD	
25 June 2009	4,800	60–75	69.3 ± 6.6	14
12 June 2010	23,200	25–35	31.3 ± 2.5	70
15 June 2011	40,000	25–35	29.4 ± 3.1	120
12 June 2012	40,000	25–35	28.7 ± 2.8	120
14 June 2013	30,000	25–35	30.2 ± 2.2	90
16 June 2014	20,000	25–35	29.5 ± 2.5	60

### Fish sampling

The composition and abundance of fish community was determined in October each year in 2007–2008 and during 2010–2014 using multi-mesh Nordic 12 gillnets (Lindeman, Replot, Finland) (stretches mesh sizes 5, 6.25, 8, 10, 12.5, 15.5, 19.5, 24, 29, 35, 43 and 55 mm, that were 30 m long and 1.5 m high). Six sampling sites were selected (three in nearshore zones and three in offshore zones), and two nets were set at each site. The 12 gill nets were set overnight (12–15 h) in the same locations every year. Fishing was conducted over three nights in each year. Catches from each net were processed separately. Captured fish were identified, counted by species, and measured for total length (TL) to the nearest mm, and body weight (BW) to the nearest 0.1 g.

Mandarin fish were quantitatively sampled by electrofishing in June from 2009 to 2014 in order to evaluate the changes of mandarin fish abundance. Electrofishing was conducted in the night (from 20:00 h to 23:00 h) along nearshore zone near to gillnet sampling sites. A total of nine sampling points (each point cover area: 2500 m^2^) were selected and fishing was conducted at three consecutive nights in each year. Catches from each point were processed separately and counted, and then measured for total length (TL) to the nearest mm, and body weight (BW) to the nearest 0.1 g.

All fish stocking and capturing procedures complied with the animal welfare laws of the Government of China and the ethical rules of the Institutional Animal Care and Use Committee of the Institute of Hydrobiology (Approval ID: Keshuizhuan 08529).

### Density and biomass estimation

The fish species that were potential prey of mandarin fish were selected for density and biomass estimation. Catch per unit effort (CUPE) in any 1 year was based on the mean CPUE of the two nets in each of the six zones. Mandarin fish density and biomass were estimated by point-sampling (nine points) electrofishing each year. The CPUE in any 1 year was based on the mean CPUE of nine sampling points. The relative fish density and biomass were determined as number per unit effort (NPUE) and biomass (g) per unit effort (BPUE), respectively.

### Size structure of forage fish

For dominant forage fish, two trap-nets were used to assess prey availability and size structure at the same time as gill nets sampling. Each trap-net set had two cod-ends, with 2 m depth and 6 mm nylon mesh. Six trap-net sets were completed in October each year. All forage fish were measured for total length (TL) to the nearest mm, and body weight (BW) to the nearest 0.1 g. We evaluated the effects of mandarin fish stocking on the size structure of dominant forage fish populations by comparing size-frequency distributions and average lengths of forage fish populations before and after mandarin fish stocking.

### Water quality

Water was sampled between 2006 and 2014 at around 10:00–12:00 hours in five different zones throughout the lake after gill net sampling in each year. Water Secchi depth was monitored *in situ* by a Secchi disk. Total nitrogen (TN) and total phosphorus (TP) were analyzed following the ‘Standard methods for the examination of the water and wastewater’^55^. Chlorophyll-*a* concentration (Chl. *a*) was determined using a fluorimeter with methanol extraction of the filtrate^56^.

### Data analysis

The differences in the fish community composition from the Biandantang Lake with respect to year were assessed by a chi-square test (χ^2^)^24^. Significant difference in biomass or density before and after mandarin fish stocking was identified using the Mann-Whitney test. The same method was adopted for Secchi depth and concentrations of total phosphorus, total nitrogen and chlorophyll-*a*. The Kolmogoro-Smirnov (K-S) test was used to examine differences in the length-frequency distribution of five dominant forage fish before and after mandarin fish stocking. A student’s t-test was used to test for the differences in the total length of five dominant forage fish before and after mandarin fish stocking. To test for differences between treatments in distribution of forage fish across the two habitat (nearshore and offshore), we used two-factor ANOVAS with treatment (pre-stocking and post-stocking) and habitat (nearshore and offshore) as the factors, and percentage (arcsine square-root transformed) of fish in each habitat as the response variable. Statistical analysis was carried out with SPSS ver. 16.0 for Windows (SPSS Inc., Chicago, Illinois). Results of statistical tests were considered significant when P < 0.05. Assumptions of normality and homoscedasticity for parametric tests were confirmed in SPSS.
